# The clinical value of the quantitative detection of four cancer-testis antigen genes in multiple myeloma

**DOI:** 10.1186/1476-4598-13-25

**Published:** 2014-02-05

**Authors:** Yao Zhang, Li Bao, Jin Lu, Kai-Yan Liu, Jin-Lan Li, Ya-Zhen Qin, Huan Chen, Ling-Di Li, Yuan Kong, Hong-Xia Shi, Yue-Yun Lai, Yan-Rong Liu, Bin Jiang, Shan-Shan Chen, Xiao-Jun Huang, Guo-Rui Ruan

**Affiliations:** 1 Peking University People’s Hospital and Institute of Hematology, Beijing Key Laboratory of Hematopoietic Stem Cell Transplantation, No.11 Xi-Zhi-Men South Street, 100044 Beijing, China

**Keywords:** Cancer-testis antigen gene, Multiple myeloma, Real-time quantitative polymerase chain reaction

## Abstract

**Background:**

Cancer-testis (CT) antigen genes might promote the progression of multiple myeloma (MM). CT antigens may act as diagnostic and prognostic markers in MM, but their expression levels and clinical implications in this disease are not fully understood. This study measured the expression levels of four CT antigen genes in Chinese patients with MM and explored their clinical implications.

**Methods:**

Real-time quantitative polymerase chain reaction (qPCR) was used to quantify the expression of MAGE-C1/CT7, MAGE-A3, MAGE-C2/CT10 and SSX-2 mRNA in 256 bone marrow samples from 144 MM patients.

**Results:**

In the newly diagnosed patients, the positive expression rates were 88.5% for MAGE-C1/CT7, 82.1% for MAGE-C2/CT10, 76.9% for MAGE-A3 and 25.6% for SSX-2. The expression levels and the number of co-expressed CT antigens correlated significantly with several clinical indicators, including the percentage of plasma cells infiltrating the bone marrow, abnormal chromosome karyotypes and the clinical course.

**Conclusion:**

MAGE-C1/CT7, MAGE-A3, MAGE-C2/CT10 and SSX-2 expression levels provide potentially effective clinical indicators for the auxiliary diagnosis and monitoring of treatment efficacy in MM.

## Background

Multiple myeloma (MM) is the second most common hematological cancer but the search for useful molecular markers in patients with MM has been highly challenging. The cancer-testis (CT) antigens are normally expressed in the testicles and trophoblastic cells of the ovary, and are also detected in some malignant tumors, such as MM and gastrointestinal stromal tumors [1-6]. CT antigens are capable of evoking spontaneous humoral and T-cell mediated immune responses and, due to their tumor-specific expression pattern and immunogenicity, CT antigens are considered as diagnostic tumor markers and potential targets for immunotherapy [7]. In addition, CT antigens are correlated with prognosis in pharyngeal cancer and non-small cell lung cancer [8,9].

Although recent studies have shown that CT antigens are widely expressed at the mRNA and protein levels in MM patients [2], and are correlated with clinical outcomes and shorter survival durations [1,10-12], the patterns of expression and clinical implications of CT antigens have not been fully characterized. Additional data on the expression levels of CT antigens in MM may provide useful information to complement recent developments in diagnostic criteria and prognostic factors, and accelerate the development of effective individualized clinical treatment strategies for MM patients.

This study aimed to measure the expression levels of four CT antigen genes, MAGE-C1/CT7, MAGE-A3, MAGE-C2/CT10 and SSX-2, in samples of bone marrow from Chinese patients with MM using real-time quantitative PCR (qPCR), and to explore the clinical implications of CT antigen gene expression levels. The results of this study strongly suggested that MAGE-C1/CT7, MAGE-A3 and MAGE-C2/CT10 expression levels could act as clinical indicators for the auxiliary diagnosis and monitoring of treatment efficacy in MM.

## Results

### The expression frequency of the four CT antigen genes in multiple myeloma

In the newly diagnosed patients, 92.3% (72/78) of specimens expressed at least one of the four CT antigen genes. The rank of the positive expression rate in the newly diagnosed MM patients was MAGE-C1/CT7 (88.5%, 69/78) > MAGE-C2/CT10 (82.1%, 64/78) > MAGE-A3 (76.9%, 60/78) > SSX-2 (25.6%, 20/78). When the samples of patients who responded to treatment (complete response, CR or partial response, PR) were included, the percentage that expressed MAGE-C1/CT7, MAGE-A3, MAGE-C2/CT10 or SSX-2 was reduced to 67.3% (76/113), 45.1% (51/113), 46.0% (52/113) and 10.6% (12/113), respectively (p < 0.05 for all). In non-responding patients, the rates of positive expression of the four genes increased significantly to 81.1% (43/53), 79.2% (42/53), 81.1% (43/53) and 32.1% (17/53), respectively (Table 1). All samples derived from the 22 healthy stem cell transplantation donors were negative for all four CT antigens.

**Table 1:** The percentage of patients with positive expression and co-expression of the four CT antigens in patients with multiple myeloma

**Samples groups**	**Percentage of patients with positive expression**				
	**MAGE-C1/CT7**	**MAGE-A3**	**MAGE-C2/CT10**	**SSX-2**	**≥1 CT antigen gene**
Newly diagnosed patients (n = 78)	69 (88.5%)	60 (76.9%)	64 (82.1%)	20 (25.6%)	72 (92.3%)
In remission (n = 113)	76 (67.3%)	51 (45.1%)	52 (46.0%)	12 (10.6%)	90 (79.6%)
Non-responders (n = 53)	43 (81.1%)	42 (79.2%)	43 (81.1%)	17 (32.1%)	52 (98.1%)
Relapsed patients (n = 12)	10 (83.3%)	9 (75.0%)	9 (75.0%)	7 (58.3%)	10 (83.3%)
*p-value	<0.01	<0.01	<0.01	<0.01	<0.01
	**Percentage of patients with positive co-expression of the four CT antigens**				
	**0 gene**	**1 gene**	**2 genes**	**3 genes**	**4 genes**
Newly diagnosed patients (n = 78)	6 (7.7%)	4 (5.1%)	14 (17.9%)	35 (44.9%)	19 (24.4%)
In remission (n = 113)	23 (20.4%)	32 (28.3%)	22(19.5%)	27 (23.9%)	9 (8.0%)
Non-responders (n = 53)	1 (2.1%)	9 (16.7%)	6 (10.4%)	23 (43.8%)	14 (27.1%)
Relapsed patients (n = 12)	0 (.0%)	0 (0%)	2 (20.0%)	3 (20.0%)	7 (60.0%)
*p-value	<0.01	0.01	0.42	<0.01	<0.01

*p-values compared with newly diagnosed patients, patients in remission and those that were non-responders to treatment using the chi-square test.

The expression frequency of all four CT antigen genes was significantly higher in patients with BM plasma cell infiltration (≥10% vs. <10%). Additionally, MAGE-A3 was expressed at a higher rate in patients with a β2-microglobulin level ≥3.5 mg/L compared to <3.5 mg/L (80.7% vs. 59.5%, p = 0.01). MAGE-A3 was also increased with ISS staging (I: 57.1% vs. II: 66.7% vs. III: 85.2%, p = 0.02) (Table 2). No significant association could be found between the expression frequency of all four CT antigen genes and age, sex, albumin or calcium in serum.

**Table 2:** Correlation of the clinicopathological characteristics of patients with MM with the expression of CT antigens

**Characteristics**		**n**	**MAGE-C1 (%)**	**MAGE-A3 (%)**	**MAGE-C2 (%)**	**SSX (%)**
Age (years)			p = 0.36	p = 0.07	p = 0.35	p = 0.19
	<60	84	81.0	64.3	71.4	29.8
	**≥**60	60	86.7	78.3	78.3	20.0
Sex			p = 0.94	p = 0.09	p = 0.57	p = 0.06
	Male	95	83.2	74.7	75.8	30.5
	Female	49	83.7	61.2	71.4	16.3
ISS staging			p = 0.19	p = 0.02*	p = 0.97	p = 0.12
	I	21	85.7	57.1	81.0	14.3
	II	42	95.2	66.7	78.6	23.8
	III	61	83.6	85.2	78.7	36.1
	Unknown	20				
BM-infiltrating plasma cells			p < 0.01*	p < 0.01*	p < 0.01*	p = 0.03*
	<10%	64	73.4	50.0	54.7	17.2
	≥10%	78	91.0	87.2	91.0	33.3
	Unknown	2				
β2-microglobulin (mg/L)			p = 0.19	p = 0.01*	p = 0.12	p = 0.52
	<3.5	37	75.7	59.5	67.6	24.3
	≥3.5	83	85.5	80.7	80.7	30.1
	Unknown	24				

*A significant association as analyzed by the chi-square test.

### Co-expression patterns of CT antigen genes in multiple myeloma

The co-expressed numbers of CT antigen genes of patients in remission decreased compared to those of untreated patients, non-responders to treatment, or relapsed patients (Table 1), which indicated that the co-expression of CT antigens was related to disease status.

We then analyzed the relationship between CT antigen expression and the clinical indices of MM. There was a moderate positive correlation between the co-expression of CT antigens and the percentage of BM plasma cell infiltration (r = 0.51, n = 244, p < 0.01). The co-expression of CT antigens was marginally correlated with the percentage of del(13q14) positive cells (r = 0.44, n = 30, p = 0.01), the percentage of del(13q14.3) positive cells (r = 0.42, n = 26, p = 0.02), β2-microglobulin levels (r = 0.27, n = 178, p < 0.01), paraprotein levels (r = 0.16, n = 164, p < 0.05), urinary free light chain levels (r = 0.21, n = 126, p = 0.02), serum calcium levels (r = 0.19, n = 199, p < 0.01), and blood sedimentation rates (ESR; r = 0.30, n = 145, p < 0.01). CT antigens were negatively correlated with hemoglobin levels (Hb: r = -0.24, n = 205, p < 0.01).

### CT antigen expression levels correlate with the percentage of plasma cells in bone marrow samples

The levels of CT antigen expression were detected by qPCR assays. The results showed that the percentages of BM plasma cell infiltration in 244 samples were positively correlated with expression levels of MAGE-C1/CT7 (r = 0.50), MAGE-A3 (r = 0.51), MAGE-C2/CT10 (r = 0.56) and SSX-2 (r = 0.23), respectively (all p < 0.01). Additionally, flow cytometry was used in 150 patients to detect the proportion of abnormal plasma cells. The expression levels of MAGE-C1/CT7 (r = 0.43), MAGE-A3 (r = 0.44), MAGE-C2/CT10 (r = 0.51) and SSX-2 (r = 0.25) were all significantly correlated with the percentages of abnormal plasma cells (p < 0.01 for all).

### CT antigen expression levels correlate with prognostic indices of multiple myeloma

Levels of β2-microglobulin, the most important and reliable prognostic factor in MM, were correlated with MAGE-C1/CT7 (r = 0.21), MAGE-A3 (r = 0.30), MAGE-C2/CT10 (r = 0.25) and SSX-2 (r = 0.20) in 178 samples (all p < 0.01).

Multivariate Cox regression analysis was performed in 107 patients who had overall survival (OS) data to compare the prognostic values of β2-microglobulin and the expression levels of the four genes. β2-microglobulin (p = 0.03), MAGE-C1/CT7 (p = 0.04) could indicate poor OS rates with a significant prognostic value.

Furthermore, the expression levels of all four genes were also correlated with Hb levels (r = -0.17 ~ -0.31, n = 205, p < 0.01) and ESR (r = -0.23 ~ -0.31, n = 148, p < 0.01). Furthermore, ages was correlated with MAGE-C1/CT7 (r = 0.24), MAGE-A3 (r = 0.29) and MAGE-C2/CT10 expression (r = 0.25) in all patients (p < 0.01 for all).

FISH analyses [including 1q21, del(13q14), del(13q14.3), 14q32 and del(17p13)] were performed in some MM patients. These have been traditionally regarded as good prognostic markers of poorer outcome [13]. In our present study, MAGE-C1/CT7 was observed to be present more frequently in patients with del(13q14) (p = 0.03). Additionally, there were higher expression levels of MAGE-C1/CT7 (p < 0.01), MAGE-A3 (p = 0.03) and MAGE-C2/CT10 (p = 0.01) in patients with del(13q14), higher levels of MAGE-C2/CT10 in patients with 1q21 (p = 0.02) and MAGE-C1/CT7 in patients with del(13q14.3) (p = 0.04). However, there were no significant differences in the expression levels of the four CT antigen genes in patients either with or without the other cytogenetic abnormalities. The expression of MAGE-C1/CT7, MAGE-A3 and MAGE-C2/CT10 in patients with 1q21, del(13q14) and del(13q14.3) exhibited an increasing trend. Nevertheless, the expression of MAGE-C1/CT7, MAGE-A3 and MAGE-C2/CT10 showed a decreasing trend in six patients with del(17p13) (Table 3), which requires further analysis. In addition, the expression levels of the four genes were significantly correlated with the percentage of del(13q14)-positive cells (MAGE-C1/CT7: r = 0.37, p = 0.03; MAGE-A3: r = 0.34, p < 0.05; MAGE-C2/CT10: r = 0.59, p < 0.01; and SSX-2: r = 0.38, p = 0.03). The above findings indicated an association between cytogenetic abnormalities and three CT antigen genes.

**Table 3:** Correlation of cytogenetic characteristics with CT antigen expression

**Characteristics**		**n**	**MAGE-C1**	**MAGE-A3**	**MAGE-C2**	**SSX**
			Level (Positive percentage)	Level (Positive percentage)	Level (Positive percentage)	Level (Positive percentage)
Del(13q14)			p < 0.01* (0.03*)	p = 0.03* (0.10)	p = 0.01* (0.20)	p = 0.25(0.15)
	No	44	214.94 ± 830.59(72.7)	8.26 ± 27.53(65.9)	2.50 ± 8.54(70.5)	0.06 ± 0.22(15.9)
	Yes	30	443.20 ± 838.11(93.3)	36.85 ± 127.23(83.3)	23.77 ± 98.61(83.3)	0.02 ± 0.06(30.0)
1q21			p = 0.13(0.68)	p = 0.11(0.10)	p = 0.02* (0.87)	p = 0.19(0.15)
	No	44	207.45 ± 644.43(79.5)	6.89 ± 24.76(65.9)	0.60 ± 1.41(75.0)	0.04 ± 0.19(15.9)
	Yes	30	454.18 ± 1050.80(83.3)	38.86 ± 127.58(83.3)	26.56 ± 98.45(76.7)	0.04 ± 0.14(30.0)
Del(17p13)			p = 0.85(0.59)	p = 0.69(1.00)	p = 0.38(1.00)	p = 0.19(0.33)
	No	68	327.33 ± 868.73(79.4)	21.57 ± 87.60(72.1)	12.08 ± 66.07(75.0)	0.05 ± 0.18(23.5)
	Yes	6	82.53 ± 118.21(100.0)	0.31 ± 0.53(83.3)	0.32 ± 0.74(83.3)	0.00 ± 0.00(0)
14q32			p = 0.79(0.94)	p = 0.67(0.05)	p = 0.68(0.42)	p = 0.74(0.86)
	No	31	251.88 ± 645.46(80.60)	34.07 ± 125.46(61.30)	20.45 ± 95.93(71.00)	0.08 ± 0.26(22.60)
	Yes	43	347.56 ± 954.95(81.40)	9.60 ± 28.30(81.40)	4.40 ± 17.23(79.10)	0.02 ± 0.07(20.90)
Del(13q14.3)			p = 0.04* (0.07)	p = 0.18(0.27)	p = 0.21(0.45)	p = 0.22(0.16)
	No	48	233.06 ± 815.48(75.00)	7.71 ± 26.40(68.80)	2.62 ± 8.19(72.90)	0.05 ± 0.21(16.70)
	Yes	26	444.86 ± 870.69(92.30)	42.25 ± 136.20(80.80)	26.83 ± 105.86(80.80)	0.02 ± 0.06(30.80)

### CT antigen expression levels correlate with the clinical course of multiple myeloma

A longitudinal analysis was performed on 58 MM patients during follow-up (median of three samples per patient; range: 2–9 samples). In order to facilitate the analysis, changes in the disease state from the clinical course of each patient were divided into three groups: improved (31 paired samples), stabilized (64 paired samples) and progressing disease (17 paired samples). The paired samples comprised samples before and after the change. Rank correlation analysis showed that the differences in the expression levels of MAGE-C1/CT7, MAGE-A3 and MAGE-C2/CT10 before and after the changes were consistent with the clinical course of individual patients (MAGE-C1/CT7: r = 0.36; MAGE-A3: r = 0.38; MAGE-C2/CT10: r = 0.28; all p < 0.01).

Patients who failed to respond to chemotherapy showed consistently high expression levels of MAGE-C1/CT7, MAGE-A3 and MAGE-C2/CT10. The increased expression levels of the CT antigens exhibited a relapse or a progressive disease, and were correlated closely with clinical variables.

**Figure 1:**
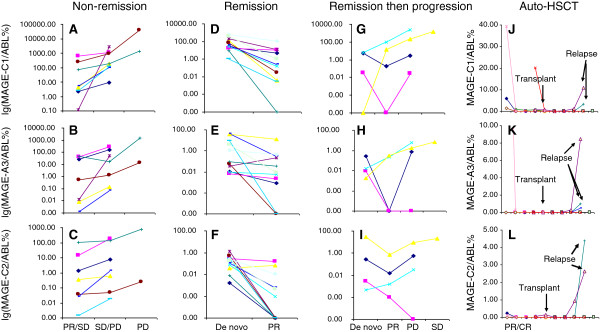
**CT antigen expression levels correlate with the clinical course of multiple myeloma.** The expression levels of MAGE-C1/CT7 **(A)**, MAGE-A3 **(B)** and MAGE-C2/CT10 **(C)** increased in eight patients who did not respond to treatment and showed non-remission courses with time. **(D, E, F)** The expression levels of the three CT antigen genes were reduced in 11 patients who responded to treatment. **(G, H, I)** Four patients with de novo disease experienced partial remission and then disease progression; the expression levels of the three CT antigen genes fell and then rose in two patients, while the other two patients showed increased expression levels before the disease progressed at later time points. **(J, K, L)** The expression levels of the three CT antigen genes were reduced or negative in 13 patients after auto-HSCT. In three patients who relapsed after auto-HSCT, one had an increased expression level of MAGE-A3, and the other two had increased expression levels of all three genes.

In contrast, patients who successfully responded to chemotherapy showed significant reductions in the expression levels of MAGE-C1/CT7, MAGE-A3 and MAGE-C2/CT10. Additionally, their numbers of BM infiltrating plasma cells, paraprotein and β2-microglobulin levels were also decreased. Patients who received auto-HSCT displayed a negative expression or reduced expression levels of MAGE-C1/CT7, MAGE-A3 and MAGE-C2/CT10, which indicated a persistent and deeper level of remission. However, one patient who relapsed after auto-HSCT showed an increased expression level of MAGE-A3, and the other two patients who relapsed after auto-HSCT exhibited increased expression levels of all three genes (Figure 1).

## Discussion

This study is the first quantitative analysis of the expression levels of four CT antigen genes, MAGE-C1/CT7, MAGE-A3, MAGE-C2/CT10 and SSX-2, in bone marrow cells from MM patients using qPCR to investigate the correlation between CT antigen expression levels and various clinical characteristics in MM. These results indicate that CT antigens are potentially effective molecular markers of MM, and have clinical implications for the auxiliary diagnosis and monitoring of treatment efficacy.

Our qPCR protocol provided a reliable and sensitive method for the quantification of MAGE-C1/CT7, MAGE-A3, MAGE-C2/CT10 and SSX-2 expression levels. The detection sensitivity was approximately 1–10 copies for plasmid DNA standards and 10^-4^ - 10^-5^ in bone marrow specimens which gave a high level of sensitivity and was less time-consuming than traditional RT-PCR [1] or immunohistochemical methods [14].

The four CT antigen genes were expressed at a high frequency in MM patients. MAGE-C1/CT7 was the most commonly expressed gene in newly diagnosed and relapsed patients, followed by the expression levels of MAGE-A3, MAGE-C2/CT10 and SSX-2. The positive expression rate of the four CT antigens was significantly lower in patients who responded to treatment, and the CT antigens were not detected in the bone marrow specimens of normal volunteers. These findings indicated that MAGE-C1/CT7, MAGE-A3, MAGE-C2/CT10 and SSX-2 were prominent MM-related tumor-specific genes, and could be used as potential molecular markers of MM.

Further analysis of the clinical data demonstrated that both the number of co-expressed CT antigens and the expression levels of MAGE-C1/CT7, MAGE-A3, MAGE-C2/CT10 and SSX-2 were associated with the percentage of plasma cells that infiltrated the bone marrow. This finding suggested that patients expressing multiple and/or high levels of CT antigens had a higher load of myeloma cells. Previous results had demonstrated that the MAGE-C1/CT7 and MAGE-A3 were expressed in isolated plasma cells using anti-CD138–conjugated magnetic beads [15], and the expression levels of both genes were associated with the numbers of CD138-positive plasma cells.

In addition, the patterns of change observed in the expression of CT antigens were consistent with the clinical course of MM. Increasing CT antigen expression indicated relapsing or progressive disease, and reduced levels or negative CT antigen expression levels occurred during treatment response. Therefore, the CT antigen expression levels could reflect the clinical status of MM, and may have clinical value for the monitoring of disease progression and evaluating of treatment efficacy.

Finally, the prognostic ability of the four CT antigens in MM was analyzed. The expression levels of MAGE-C1/CT7, MAGE-A3, MAGE-C2/CT10 and SSX-2 were correlated with prognostic factors for MM, including β2-microglobulin, abnormal chromosome karyotypes, age and the hemoglobin level. It appeared that MAGE-C1/CT7 had a similar prognostic value to β2-microglobulin, which is currently the most valued prognostic indictor for MM. Accordingly, MAGE-C1/CT7 was identified as the leading candidate as a prognostic indictor compared to the other three genes. Chromosomal abnormalities, including 1q21, del(13q14) and del(13q14.3), were associated with poor survival rates and a more aggressive disease course [13]. In the present study, higher expression levels of MAGE-C1/CT7, MAGE-A3, MAGE-C2/CT10 were found in patients with del(13q14), higher levels of MAGE-C2/CT10 were found in patients with 1q21 and MAGE-C1/CT7 was more commonly found in patients with del(13q14.3), which indicated that the three genes had the same negative effect on prognosis. Del(17p13) was also found to be an important molecular cytogenetic marker for poor prognosis. The expression of MAGE-C1/CT7 has been reported to be more frequent in patients with del(17p13) (p = 0.047) [16]. MAGE-C1/CT7, MAGE-A3 and MAGE-C2/CT10 showed a decreasing trend in six patients with del(17p13), but the correlation between CT antigen gene expression and del(17p13) require further validation in a large population of patients.

The correlations between MAGE-C1/CT7, MAGE-A3, MAGE-C2/CT10 or SSX-2 expression and clinical indicators in the present study suggest that each CT antigen has a unique role in MM. The expression of MAGE-C1/CT7 was the most useful indictor in patients with MM, while MAGE-A3 and MAGE-C2/CT10 might confer an additive value for the prediction of disease complications and prognosis. A previous study showed that MAGE-A3 was also expressed in patients with MGUS and myeloma, which suggested that the expression of MAGE-A family members could be a common phenomenon or perhaps even an early event in the evolution of plasma-cell diseases [15]. SSX-2 was expressed at very low levels in MM patients. Nevertheless, the positive expression of SSX-2 in patients with MM may still have a value for the diagnosis and prognosis of MM patients. It had been reported previously that SSX-2 levels were significantly associated with an adverse prognosis and reduced survival [17], and future studies will define the role of SSX-2 expression in MM pathogenesis.

## Conclusion

MAGE-C1/CT7, MAGE-A3, MAGE-C2/CT10 and SSX-2 were found to be frequently expressed in MM patients. The positive expression of CT antigens, their co-expressed numbers, and the expression levels were significantly associated with recognized clinical indicators that reflect the severity of the disease, including BM plasma cells, β2-microglobulin and abnormal chromosome karyotypes. The expression levels of MAGE-C1/CT7, MAGE-A3 and MAGE-C2/CT10 were also correlated with clinical course. The expression levels of the four CT genes could be potentially used as clinical indicators for the auxiliary diagnosis and monitoring of treatment efficacy in MM patients. To improve the diagnosis and treatment of MM patients, the roles of CT antigens in the development and progression of this disease await clarification.

## Methods

### Patients

Between November 2006 and December 2010, 144 MM patients were enrolled in this study; 78 (54.2%) patients were newly diagnosed, 17 (11.8%) had a complete response (CR), 24 (16.7%) had a partial response (PR), five (3.5%) had a progressive disease (PD), 10 (6.94%) had stable disease (SD) and 10 (6.94%) were from patients with relapsed disease. The median patient age was 58 years, and 66.0% of patients were male. The clinical data of all patients is showed in Table 1.

The most common isotype was IgGκ 44 (30.6%), 29 (20.1%) were IgGλ, 14 (9.7%) were IgAκ, 18 (12.5%) were IgAλ, 6 (4.2%) were IgDλ, 18 (12.5%) were κ light chain isotype, 11 (7.6%) were λ light chain isotype and four (2.8%) patients had nonsecretory MM. Most patients after diagnosis accepted four cycles of induction treatments that included BD (consisting of intravenous bortezomib at 1.0 or 1.3 mg/m^2^ on days 1, 4, 8, and 11, 40 mg dexamethasone on days 1 to 4, followed by a 3-week rest period) or TD (thalidomide given orally at 200 mg/d; and dexamethasone given orally at 40 mg on days 1 to 4, followed by a 4-week rest period). The therapeutic response was assessed after induction treatments. If PR occurred, patients proceeded to autologous hematopoietic stem cell transplantation (auto-HSCT). Both transplanted and non-transplanted patients received maintenance therapy with thalidomide (75–200 mg/d) plus dexamethasone (20–40 mg/d) for 1 year. Diagnosis, staging and therapeutic effects were defined using widely used standard criteria [18,19]. Informed consent was obtained from all patients and 22 allogeneic stem cell transplantation donors prior to their enrollment in the study. The study design adhered to the principles of the Helsinki Declaration and was approved by the ethics committee of Peking University People’s Hospital.

### Bone marrow samples and sample preparation

Two-hundred and fifty-six BM samples were obtained from patients with MM during routine diagnostic procedures at the Peking University People’s Hospital. Mononuclear cells were isolated from BM samples using standard Ficoll-Hypaque density gradient centrifugation. RNA was extracted from mononuclear cells using the TRIzol technique (Invitrogen, Carlsbad, CA, USA) according to the manufacturer’s instructions. cDNA was synthesized as previously described [20].

### Real-time quantitative PCR

The primers and probes were designed using Primer Express 2.0 software (Applied Biosystems, Foster City, California, USA) as follows: ABL (forward 5′-CCGCTGACCATCAATAAGGAA-3′, reverse 5′-GATGTAGTTGCTTGGGACCCA-3′ and probe 5′-FAM-CCATTTTTGGTTTGGGCTTCACACCATT-TAMARA-3′) [21], MAGE-C1/CT7 (forward 5′-TTGTCTTCTGGGAACCTTGACTC-3′, reverse 5′-TGAGGGACACATACATCCTAAAAGC-3′ and probe 5′-FAM-ACTGCCTGGGCCTCCTCTGCTGT-BHQ-3′), MAGE-A3 (forward 5′-GGTGAGGAGGCAAGGTTCTGA-3′, reverse, 5′-GTGCTGACTCCTCTGCTCAAGAG-3′ and probe, 5′-FAM-AGATCTGCCAGTGGGTCTCCATTGCC-BHQ-3′), MAGE-C2/CT10 (forward 5′-GTGTGAGGCACACAGCCTAAAG-3′, reverse 5′-GGAGGCATGACGACTTCTTCA-3′ and probe 5′-FAM-AGGAGTCAAGGCCTGTTGGATCTCATCA-BHQ-3′) and SSX-2 (forward 5′-TAACCGTGGGAATCAGGTTGA-3′, reverse 5′-CCTCCGAATCATTTCCTTCCT-3′ and probe 5′-FAM-CCGAAGATCATGCCCAAGAAGCCAG–BHQ-3′). The 10-μl PCR reaction mixture contained 5 μl 1× TaqMan® Universal PCR Master Mix (Applied Biosystems, Foster City, California, USA), 400 nM primers, 250 nM fluorescent probes and 150–500 ng cDNA. PCR was performed using the ABI PRISM® 7500 FAST Sequence Detection System (Applied Biosystems) at 50°C for 2 min and 95°C for 10 min, followed by 50 cycles at 95°C for 15 s and 60°C for 1 min. The expression levels of the four CT antigen genes (MAGE-C1/CT7, MAGE-C2/CT10, MAGE-A3 and SSX-2) were quantified by qPCR using the abelson (*ABL*), glyceraldehyde-3-phosphate dehydrogenase (*GAPDH*), beta-glucuronidase (*GUS*) and β2-microglobulin (β*2M*) genes as internal control genes, respectively, as described previously [21,22]. On the basis of our previous study of the four control genes, *ABL* was a more appropriate control gene in MM patients (*Clinical and Experimental Medicine*, accepted, DOI:10.1007/s10238-013-0257-2, 2013). Therefore, the copy numbers of the four CT antigen genes and *ABL* were calculated using the C_t_ values on standard curves, and results were analyzed following the guidelines proposed by the European Study Group for qPCR [23].

Serial dilutions of the plasmids (10^6^, 10^5^, 10^4^, 10^3^, 10^2^, 10^1^ and 10° copies) that expressed ABL and the four CT antigen genes were amplified by qPCR to construct standard curves for quantification, respectively. The plasmids were prepared as previously described ^18^. A linear correlation was observed between the C_t_ values and plasmid copy number, with a correlation coefficient of >0.99 for all curves. The standard curves revealed a similar efficiency of amplification for ABL, MAGE-C1/CT7, MAGE-A3, MAGE-C2/CT10 and SSX-2, with slopes of -3.22, -3.28, -3.57, -3.43 and -3.51, respectively. As the amplification efficiency was similar for all genes, the CT antigens were quantified against the ABL standard curve in bone marrow specimens to decrease experimental error. For each measurement, the curve threshold amplification was set at 0.08 for ABL and the other three CT antigen genes. The detection sensitivity was approximately 1–10 copies in the plasmid DNA standards and 10^-4^ - 10^-5^ in CT antigen-positive bone marrow specimens.

### Flow cytometry

Fresh BM samples were analyzed using standard flow cytometry using a four-color immunofluorescence technique as previously described [24]. BM samples were analyzed for the surface and cytoplasmic staining of CD19, CD38, CD45, CD56, CD117, CD138, κ and λ, amongst others, to identify malignant plasma cells.

### Cytogenetics

Cytogenetic FISH analyses were performed using standard methods [25]. The definition of a cytogenetic clone and the karyotype descriptions followed the International System for Human Cytogenetic Nomenclature.

### Statistical analysis

All statistical analyses were performed using SPSS 13.0 (SPSS Inc., Chicago, IL, USA). Analysis of variance was used for continuous variables and χ^2^ tests were used for categorical variables. The correlation between the clinical indices and the expression level of the four CT antigen genes were analyzed using linear correlation analysis. Multivariate Cox regression analysis was performed to compare the prognostic value. Rank correlation analyses were used to assess the correlation between the clinical course and the expression levels of the CT antigen genes. P-values <0.05 was considered significant for all analyses.

## Competing interest

The authors declare that they have no competing interest.

## Authors’ contributions

GRR and XJH designed the project, advised on the study and revised manuscript. YZ performed RQ-PCR experiment and all statistical analyses, contributed to the interpretation of the data and wrote the manuscript. YZ, LB, JL, KYL, HC, YK, HXS, BJ, and SSC fulfilled ethical authorizations, collected and stored clinical data. JLL, YZQ and LDL performed sample handling and storage. YRL and YYL contributed to flow cytometry and cytogenetics detection, respectively. All authors read and approved the final manuscript.
